# Central Scotoma

**Published:** 1881-09-20

**Authors:** A. G. Hobbs

**Affiliations:** Prof. of Diseases of Eye, Ear and Throat, in Southern Medical College, Atlanta, Ga.


### CENTRAL SCOTOMA.

BY A. G. HOBBS, M. D., PROF. OF DISEASES OF EYE, EAR AND THROAT, IN SOUTHERN MEDICAL COLLEGE, ATLANTA, GA.

During the past four months I have had under my care two cases of this rather rare form of ambliopia, and they have proved quite interesting to myself because of the gradual but very perceptible improvement of both cases under persistent treatment.

Case 1. A man æt 45, came to my office with phlyctenular conjunctivitis caused by the irritation produced by cinders. After a ten days treatment, his eyes were apparently well, but he complained of poor vision—inability to read or recognize a friend across the street. I first thought it due to the atropia, but the bad vision continued, and neither M nor H, glasses improved it. Vision 20-200 when looking directly at the test type, but became 20-100 when looking three feet to either side. In order to read No. 16 Jasger at 18 inches, he looked at the margin of the page.

An ophthalmoscopic examination showed the disk to be decidedly congested, and together with the side of the disk next to the macula lutea slightly swolen and hazy. The patient was an inveterate smoker and somewhat addicted to alcohol.

I stopped both, applied electricity and gave 1-10 gr. strychnia daily. After two weeks ophthalmoscopic examination showed paleness of the disk next the yellow spot; his defect of vision was much more apparent at mid-day, during which time he was compelled to obstruct the bright rays with blue glasses. Pupils normal, not even sluggish. He could distinguish all colors but green and red. His greatest defect was limited to or at least was greatest at the central part of the field lying between the yellow spot and the optic disk. A test of his vision now showed 20-70 by looking one foot to either side of the test type. After three months' treatment his vision was brought up to 20-30 direct, and No. 6 Jasger at 18 inches, at which point it still remains. Ophthalmoscopic examination with—1 D, (observer's vision being E,) shows nothing abnormal unless, perhaps, a slight grayish aspect to the field.

Case 2. Male, æt 33, presented all the symptoms of Case 1, with the addition of a syphilitic history, the central scotoma was not so plainly marked either with the ophthalmoscope or test type, and vision only ran down as low as 20-100 even when looking directly at the type, and not quite so low when he directed his vision one foot to either side. Case 2 was also a great smoker.

By stopping his cigars and putting him on the same treatment as Case 1, together with iodide of potassium his vision improved in less than three months to normal.

This is considered one of the rare manifestations of ambliopia and its pathology is still in obscurity. Upon the first examination with the ophthalmoscope it would seem that there existed an inflammatory condition of the ocular and of the optic nerve and the surrounding part of the disk next the yellow spot, but after a few weeks or months the appearances are more like atrophy than inflammation. Only three previous cases have fallen under my notice of this tobacco form of ambliopia showing a central imperfection of vision. These fourth and fifth cases have each been restored to more perfect vision than the first three. I attribute the success in these two latter cases to the vigorous use of the galvano-faradic current with a water-cup electrode.

I could trace no heredity in either of these cases. Most observers think tobacco the sole cause, and recommend its disuse only as necessary in the treatment. Through a misunderstanding case No. 1 was not under treatment for about four weeks, thinking, as he expressed it, "that if tobacco caused it, its disuse would cure it," but his vision did not improve during those weeks, and improvement was perceptible almost immediately after he began a systematic course of treatment.

Case 2d began treatment coincident with his disuse of tobacco, and his improvement continued till his vision became normal.

I have recorded these two cases to show the good results of the treatment instituted rather than to claim for it anything original.

